# Functionalized biochar-supported magnetic MnFe_2_O_4_ nanocomposite for the removal of Pb(ii) and Cd(ii)

**DOI:** 10.1039/c8ra09061k

**Published:** 2019-01-02

**Authors:** Lianke Zhang, Jinyue Guo, Xuemin Huang, Weida Wang, Peng Sun, Yumei Li, Jianhong Han

**Affiliations:** School of Environmental and Municipal Engineering, Xi'an University of Architecture and Technology Xi'an 710055 PR China lkzhang@126.com nkdlkzhang@163.com +86-472-5951568 +86-472-5951657; School of Energy and Environment, Inner Mongolia University of Science and Technology Baotou 014010 PR China

## Abstract

In this study, a novel magnetic biochar-MnFe_2_O_4_ nanocomposite (BC/FM) was prepared using low-cost corn straw and MnFe_2_O_4_ by sol–gel/pyrolyzing route using egg white, which has abundant functional groups (–NH_2_ and –COOH). Following that, its composition, morphology and structure was characterized by various techniques including SEM-EDX, BET, XRD, and VSM. Batch experiment of the adsorption for Pb(ii) and Cd(ii) including influence of pH, kinetics, isotherm and thermodynamics was also studied. The results demonstrated that biochar could effectively support MnFe_2_O_4_, which displayed high dispersion on the surface of the biochar and possessed abundant functional groups and high surface area contributing to superior performance on Pb(ii) and Cd(ii) removal. Therein, MnFe_2_O_4_ with high magnetism is convenient for separating the magnetic BC/FM from an aqueous medium. Adsorption experiment results indicate that Pb(ii) and Cd(ii) removal by BC/FM was closely related to pH with the best value of pH 5.0, and the process reached equilibrium in 2 h. The adsorption process is well-described by the pseudo-second-order kinetic model and Sips (Freundlich–Langmuir) model. Thermodynamic studies suggest that the adsorption process is spontaneous and exothermic. The maximum experimental adsorption capacity of BC/FM is 154.94 and 127.83 mg g^−1^ for Pb(ii) and Cd(ii), respectively, in single-solute system, which is higher than that of some of the other adsorbents of biochar or biochar-based composites. In bi-solute system, the preferential adsorption order of BC/FM for the two metals is Pb(ii) prior to Cd(ii). Finally, FTIR and XPS analysis verified that the main mechanism of Pb(ii) and Cd(ii) removal by BC/FM is by forming Pb/Cd–O or complexation of carboxyl and hydroxyl and ion exchange. Therefore, the prepared magnetic BC/FM composite, as an excellent adsorbent, exhibited potential applications for the removal of Pb(ii) and Cd(ii) from wastewater.

## Abstract 2

In this study, a novel magnetic biochar-MnFe_2_O_4_ nanocomposite (BC/FM) was prepared using low-cost corn straw and MnFe_2_O_4_ by sol–gel/pyrolyzing route using egg white, which has abundant functional groups (–NH_2_ and –COOH).

## Graphical abstract

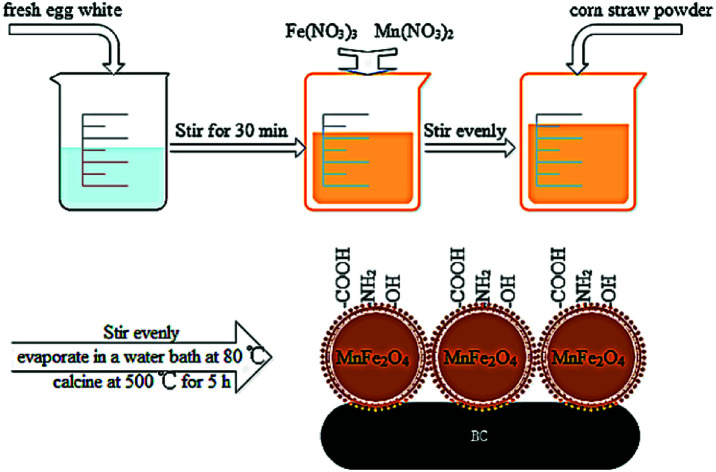

## 1. Introduction

In the past decades, with the rapid pace of economic development, increasing amount of heavy metal ions such as lead and cadmium were released into the environment, which then entered the human body through various ways, leading to severe health issues in human beings.^1,2^ As one of the toxic heavy metals, lead can cause many diseases related to digestive, circulatory, respiratory and other systems, and even death when it enters the human body.^3,4^ As for another heavy metal ion – cadmium, it can accumulate in the body and cause diseases of the lung, bone and kidney.^5,6^ Comparing with their presence alone, the coexistence of lead and cadmium will pose a higher hazard to environmental pollution and human health. Therefore, it is essential to develop promising techniques for simultaneous removal of Pb(ii) and Cd(ii) from wastewater prior to their discharge into the environment.

So far, various methods including chemical precipitation, electrochemical processes, ion exchange, and adsorption have been developed to deal with heavy metals from wastewater.^7–10^ Among these technologies, adsorption is a relatively advantageous method for water treatment processes because of low cost, simple operation and high efficiency.^10–12^ A variety of adsorbents for wastewater treatment have been developed. Biochar (BC) is a pyrolytic carbonaceous organic matter that is often produced under an oxygen-limited condition from waste biomass, which is cheap and easy to obtain. Due to its characteristic porous structure, high specific surface area, abundance of functional groups and a high ion exchange capacity, BC was chosen as a well-suited host material that can stabilize and disperse nanoparticles to further enhance the adsorption capacity of the composite materials. Moreover, BC has been deemed to be an ideal material for the removal of metal ions from an aqueous medium.^13,14^ Previous studies have suggested that biochars derived from different biomass can remove heavy metals, for instance Pb and Cd, effectively.^15–18^ However, unprocessed biochars usually have lower adsorption ability for metal ions than activated carbon. In addition, the separation and recycling from aqueous solutions after adsorption would be hard to achieve.^3,13^ These disadvantages have become an obstacle to their practical application. To solve these problems, the preparation of magnetic biochar composites through the introduction of magnetic particles has become a research hotspot in recent years.^13,14^ Magnetic biochar composites have good separation performance from water under an external magnetic field. Besides, the enhancement of removal efficiency for metal ions has been experimentally verified.^19–22^

Various types of magnetic particles, such as iron oxide (Fe_2_O_3_, γ-Fe_2_O_3_, and Fe_3_O_4_),^13,21^ ferromanganese binary oxide,^22^ magnetic gelatin^14^ and iron oxy-hydroxides,^23–25^ were used to achieve magnetic properties in biochar materials. Manganese ferrospinel MnFe_2_O_4_, however, exhibits outstanding magnetic property and structural stability due to the change in valence state of iron. Moreover, the magnetic MnFe_2_O_4_ material has been proved to be an outstanding adsorbent for metal ions in the aqueous medium.^26,27^ Due to the active functional carboxyl and hydroxyl groups on the surface allowing chemical interactions, the MnFe_2_O_4_ usually has a large adsorption capacity for metal ions. The sorption abilities of MnFe_2_O_4_ for removing Pb(ii) from the aqueous solutions have been reported. However, MnFe_2_O_4_ exhibits several disadvantages, such as weak mechanical strength, ease of agglomeration and low removal efficiency. Accordingly, in recent years, the development of magnetic composite materials with MnFe_2_O_4_ has attracted wide attention.^28–30^ Motivated by these observations, we developed a new magnetic composite comprising of MnFe_2_O_4_ and biochar (BC) for metal ions removal from aqueous medium with high efficiency. When the MnFe_2_O_4_ is combined with biochar, the obtained composite is expected to show both good adsorption performance and magnetic property, and to maximally utilize the benefits of both MnFe_2_O_4_ and BC for heavy metal removal.

In this study, magnetic MnFe_2_O_4_ particles were successfully immobilized on biochar by the sol–gel/pyrolyzing route using egg white that has abundant functional groups (–NH_2_ and –COOH). It not only overcomes the problem of biochar recovery but also stops the aggregation of MnFe_2_O_4_ and improves the adsorption ability of contaminants. This study was designed to elucidate the respective roles of biochar and MnFe_2_O_4_, and evaluate the performance of BC/FM for the removal of Pb(ii) and Cd(ii) from the coexistence system, which is different from previous studies on heavy metal removal from single-component batch solution using biochar-based composites. Their adsorption behavior was investigated through characteristic and batch adsorption experiments. Their adsorption mechanisms were further investigated. It is of great social significance to control pollution and reduce emission of heavy metal wastewater. At the same time, it is of great significance to seek new ways for maize straw waste resource utilization and promote agriculturally sustainable development of low carbon material loop.

## 2. Materials and methods

### 2.1 Chemical reagents

All chemicals used in this experiment were of analytical grade. Pb(ii) and Cd(ii) stock solutions were obtained by dissolving Pb(NO_3_)_2_ and Cd(NO_3_)_2_ in deionized water. Different concentrations of Pb(ii) and Cd(ii) working solutions were prepared by diluting the above stock solutions before use.

### 2.2 Preparation of adsorbents

**Fig. 1:**
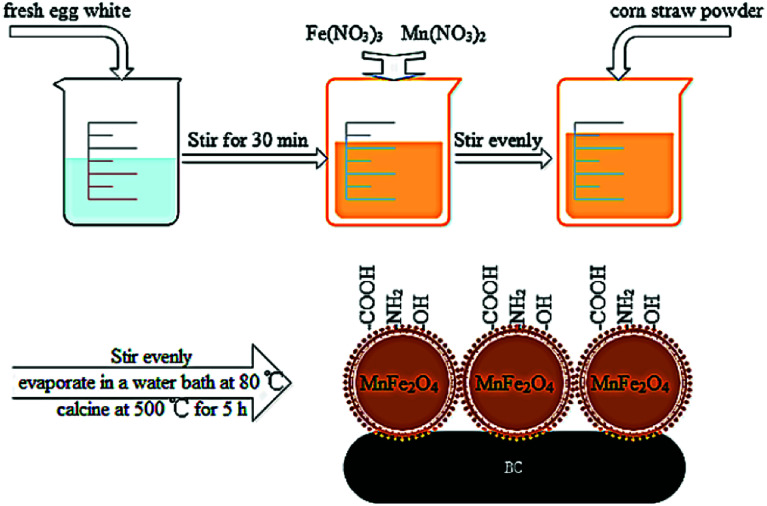
Schematic illustration of the preparation processes of BC/FM.

BC/FM was prepared through sol–gel/pyrolyzing route using corn straw as substrates and the detailed steps are as follows. Firstly, a homogeneous solution was prepared by stirring 60 mL egg white for 30 min. Then, 8.013 g Fe(NO_3_)_3_·9H_2_O and 4.7 mL Mn(NO_3_)_2_ were slowly added into the above homogeneous egg white solution and stirred vigorously.^31^ 5 grams corn straw (ground to pass 1 mm sieve) was added in the mixed solution and stirred evenly, evaporated in an 80 °C water bath. Subsequently, in nitrogen atmosphere, the mixed solution was pyrolyzed for 5 h in a muffle furnace at 500 °C, and then cooled. Composite material preparation was completed, the material was named BC/FM. The obtained BC/FM was ground to pass 0.15 mm sieve and was washed using deionized water until reaching neutrality, and were then dried. Bare MnFe_2_O_4_ nanoparticles were synthesized by a similar process in the absence of corn straw. A schematic diagram of the BC/FM fabrication is shown in Fig. 1.

### 2.3 Characterization of samples

The morphologies of MnFe_2_O_4_, BC and BC/FM were studied using scanning electron microscopy, energy dispersive X-ray spectroscopy (SEM-EDX, Quanta 400), and high resolution transmission electron microscopy (HRTEM, FEI Tecnai G2 F30). The specific surface area was examined based on nitrogen adsorption experiments (BET, Builder SSA-4200). X-ray diffraction measurement was performed using X-ray diffractometer (XRD, Philips APD 3720). The samples were scanned in the range of 2*θ* from 10° to 80° with a 0.02° step at a scanning speed of 4° min^−1^. The magnetization of BC/FM was determined using SQUID magnetometer at 700 K. The surface functional group of the samples was evaluated using Fourier transform infrared spectroscopy at a resolution of 2 cm^−1^ (FTIR, Nicolet 6700). X-ray photoelectron spectrometer (XPS, ESCALAB 250Xi) was used to analyze the surface chemical composition of BC/FM before and after adsorption of Pb(ii)/Cd(ii).

### 2.4 Adsorption experiments

Adsorption experiments were performed in a 150 mL conical flask with stirring at 150 rpm to study the adsorption effects of Pb(ii) or Cd(ii) by BC/FM. The experiment of pH influence on adsorption was carried out in the range of pH 2–8 using 0.1 M NaOH and HCl solutions. For the adsorption kinetics, 50 mg BC/FM was added to 40 mL Pb or Cd solutions, and aliquots were sampled at different time intervals (1–300 min). The adsorption isotherm experiments in the single solute system were carried out at three temperatures of 25 °C, 35 °C and 45 °C, and for the bi-solute system, the temperature was 25 °C. After adsorption, the mixtures were filtered by 0.22 μm filters and then the concentration of the metal ions were analyzed by an atomic absorption spectrophotometer (PE-AA800). All experiments were performed in triplicate. Adsorption capacity and removal rate were calculated according to the following equations:1
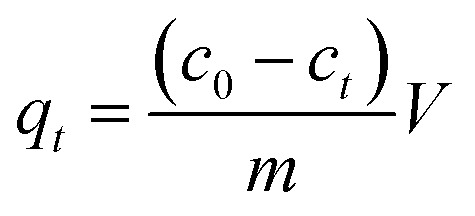
2
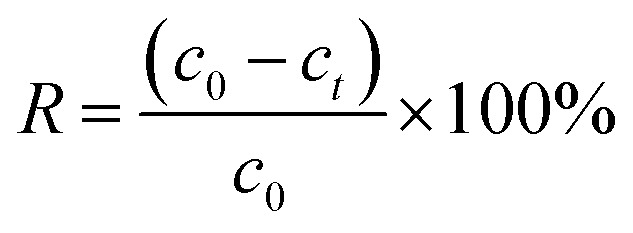
where *q*_*t*_ is the adsorption quantity at time *t* (mg g^−1^); *c*_0_ is the initial concentration (mg L^−1^) in the pre-adsorption solution; *c*_*t*_ is the instantaneous concentration (mg L^−1^) at time *t* (min); *V* is the sample volume (L); *m* is the dosage of BC/FM (g); *R* is the removal rate.

## 3. Results and discussion

### 3.1 Adsorbent characterization

**Fig. 2:**
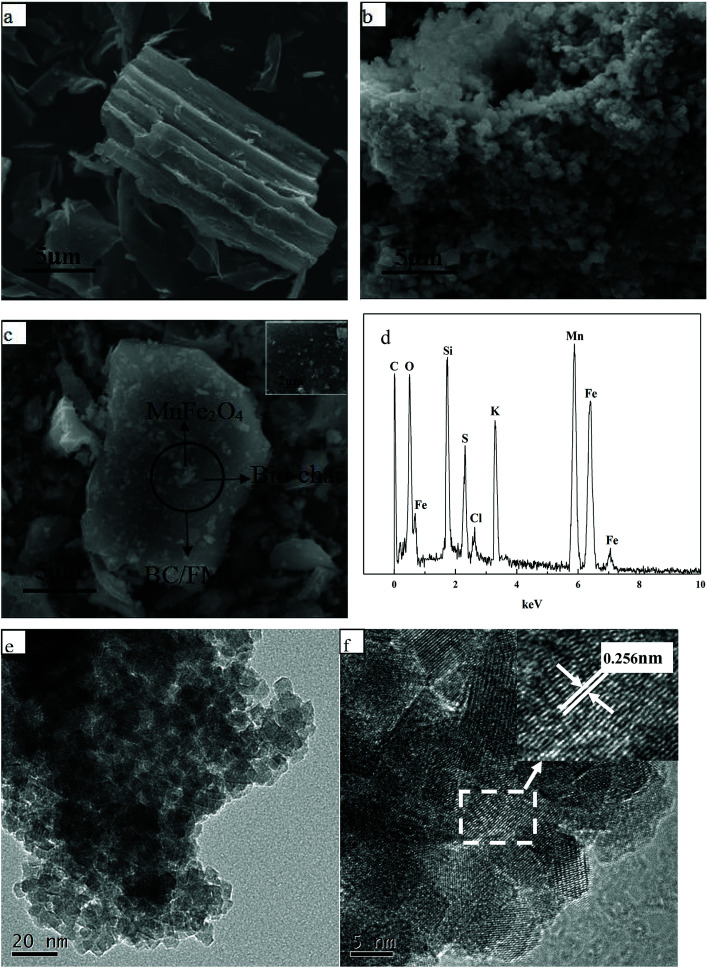
SEM image of (a) BC, (b) MnFe_2_O_4_, (c) BC/FM; (d) EDS analysis of BC/FM; (e) TEM image and (f) HRTEM image of BC/FM.

The surface morphology and chemical composition of BC, MnFe_2_O_4_ and BC/FM were characterized using SEM-EDX, TEM and HRTEM, and typical images are given in Fig. 2. The surface of biochar derived from corn straw was smooth and porous without impurities as seen in Fig. 2a. As shown in Fig. 2b, the prepared MnFe_2_O_4_ exhibited irregular sponge-like bulk particles with significant aggregation, which led to the decline in the adsorption effect.^28^ In comparison, the spongiform MnFe_2_O_4_ particles for BC/FM composite were uniformly dispersed and loaded onto the biochar surface as in Fig. 2c. It can be seen that the biochar effectively prevents the agglomeration of MnFe_2_O_4_ particles, and ensures that the BC/FM composite still has a large surface area.^32^ Moreover, the EDX indicated the presence of C, O, Fe and Mn in the BC/FM composite (Fig. 2d). The peaks of Fe and Mn further show that MnFe_2_O_4_ was loaded onto the biochar framework. The peak of C confirms the existence of biochar, while the peak of O was ascribed to both MnFe_2_O_4_ and the oxygen-containing functional group of biochar. Fig. 2e and f exhibits the TEM and HRTEM images of a typical sample of BC/FM nanoparticles. It is displayed that the samples are homogeneous square or polygon shaped particles. The average particle size is lower than 10 nm. The HRTEM image exhibits well defined fringes as shown in Fig. 2f, and the inset expresses the enlarged pattern of the dotted box, which shows that the interplanar spacing is 0.256 nm and can be attributed to the (311) planes of MnFe_2_O_4_.

**Fig. 3:**
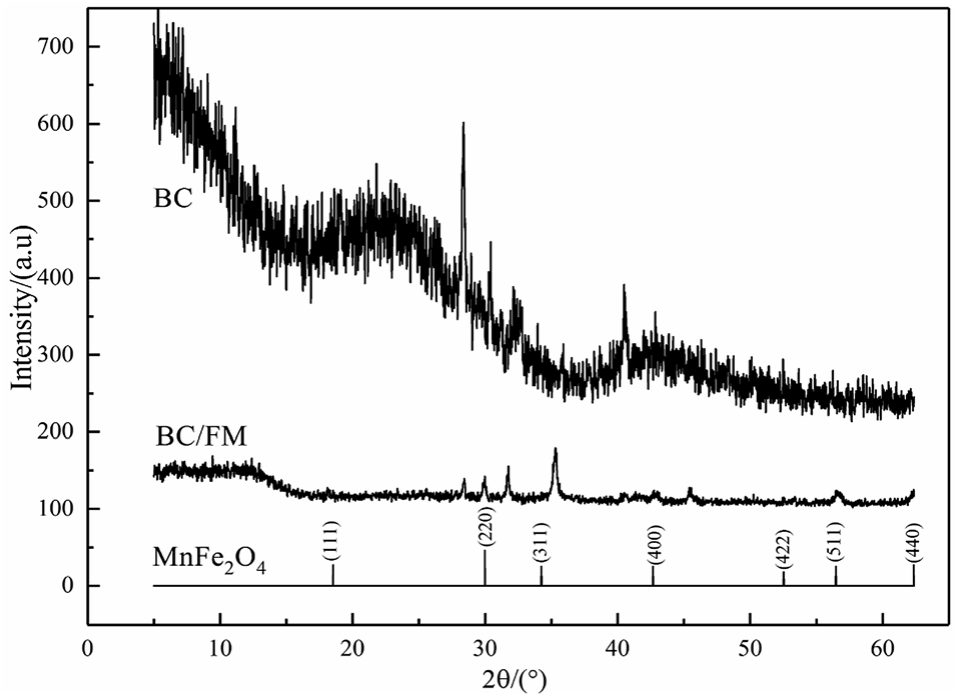
XRD pattern of BC/FM.

The XRD pattern is shown in Fig. 3. For BC, the wide peak located at about 24° represents a typical diffraction pattern of amorphous carbon.^33^ The expected spinel iron ferrite structure is present in the XRD patterns of BC/FM, which proves that MnFe_2_O_4_ exists in BC/FM. The diffraction peaks are located at 30.20°, 35.6°, 43.24°, 57.26° and 62.9° (as major peaks), which agrees with the crystal planes (220), (311), (400), (422) and (440) of MnFe_2_O_4_ (JCPDS: 38-0430).^34^ The additional peaks located at about 32° and 45° in BC/FM are assigned to NaCl (PDF number 00-005-0628) in biochar ash.

**Fig. 4:**
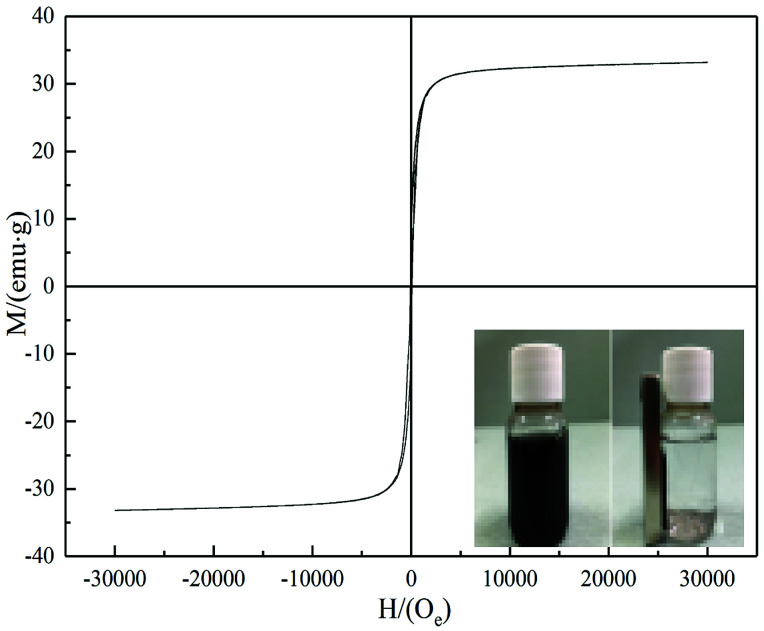
*B*–*H* curve of BC/FM. The inset image shows the attraction to a permanent magnet.

The characteristics of magnetization curves determine the practicability of ferromagnetic materials.^35^ Magnetic properties of BC/FM were determined using a vibrating sample magnetometer (Fig. 4), and its magnetization saturation (*M*_s_) was 33.188 emu g^−1^. At 700 K, coercivity (*H*_c_) and remnant magnetization (*M*_r_) were 86.389 Oe and 7.866 emu g^−1^. According to the experimental results, BC/FM dispersed in an aqueous medium was separated within 20 seconds by an external magnetic field of 20 mT. These results suggest that BC/FM has outstanding magnetic property, which is conducive to the separation of adsorption and desorption regeneration.

The BET test results showed that specific surface area of BC/FM was 38.05 m^2^ g^−1^, which is greater than those of pure MnFe_2_O_4_ (2.68 m^2^ g^−1^),^36^ and sponge-like porous MnFe_2_O_4_ (29.80 m^2^ g^−1^).^31^ The significant increase in BET surface area was expected to improve the adsorption process of both the metals.

### 3.2 Effect of pH

**Fig. 5:**
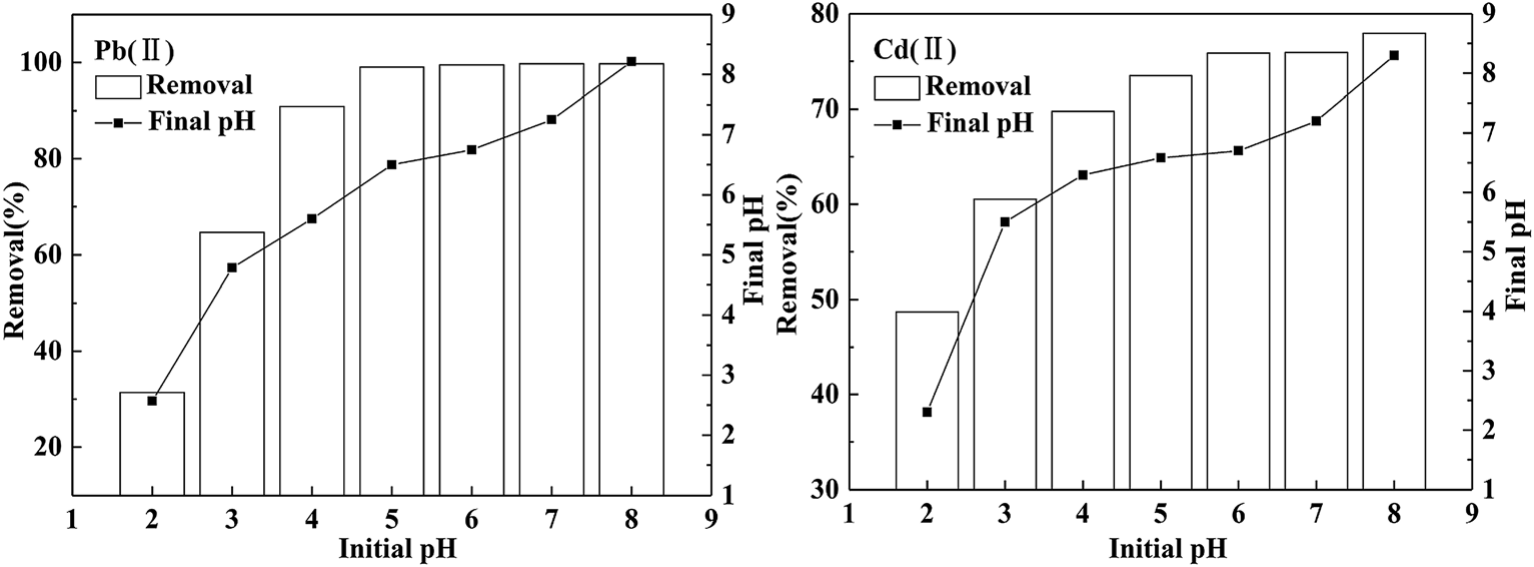
Effect of pH on adsorption of Pb(ii) and Cd(ii) on BC/FM. Conditions: *C*_0_(Pb(ii)) = 100 mg L^−1^, *C*_0_(Cd(ii)) = 20 mg L^−1^, BC/FM dose = 1.25 g L^−1^, temperature = 298 K, and *t* = 5 h.

In this study, the influence of pH on the adsorption process is mainly manifested in two aspects: properties of BC/FM and speciation of two metals. The effect of pH on the adsorption by BC/FM of both the metals is shown in Fig. 5. To exclude the interference of precipitation, the experimental pH range of 2–8 was selected.^35^ As shown in Fig. 5, for Pb(ii) and Cd(ii), the adsorption process is evidently related to the solution pH, and the removal rate increases with the pH from 2 to 8. At pH 2.0, the removal percentages of both the metal ions were low. For Pb(ii), the removal percentage increased evidently and reached 90.9% as the solution pH increased from 2 to 4; it reached 99.0% when the pH increased to 5.0. The removal percentage remained stable between pH 5 to 8. In case of Cd(ii), when the pH increased from 2 to 5, the removal percentage showed a gradual increase. The removal rate reached 73.5% at pH 5.0, and a small increase after pH 5.0 was observed.

On the one hand, pH is a significant environmental factor directly related to the surface nature of BC/FM. The effect of pH on the surface characteristics of BC/FM was derived from two components, namely, BC and MnFe_2_O_4_. Heavy metals were bound to BC mainly through ion exchange and complexation with functional groups, for instance carboxyl and hydroxyl group of BC.^37–39^ MnFe_2_O_4_ mainly bind heavy metals by complexation.

3 2R_1_COO_(S)_^−^ + M_(aq)_^2+^ ⇔ [(R_1_COO^−^)_2_M^2+^]_(S)_

4 2R_1_O_(S)_^−^ + M_(aq)_^2+^ ⇔ [(R_1_O^−^)_2_M^2+^]_(S)_

5 2R_2_OH_(S)_ + M_(aq)_^2+^ ⇔ [(R_2_O^−^)_2_M^2+^]_(S)_ + 2H^+^

where R_1_ and R_2_ denotes the surface of BC and MnFe_2_O_4_, respectively. Therefore, low pH is not conducive to bind heavy metals by both BC and MnFe_2_O_4_ due to the competing H^+^. Besides, due to the presence of MnFe_2_O_4_, BC/FM is mainly composed of surface hydroxyl groups. At lower pH, BC/FM surface is positively charged and repels metal ions. When the pH increases, the adsorption sites become negatively charged, and more metal cations could be removed. The experimental results shown in Fig. 5 indeed confirm the above explanation.

On the other hand, speciation of metal ions is also affected by the solution pH.^35,40^ According to the speciation of Pb and Cd at different pH, the removal of both the metals is explained further. At solution pH 2–5, the main species of lead is Pb(ii).^40^ Therefore, free lead can be adsorbed on BC/FM. When pH is above 6, formation of PbOH^+^ and Pb(OH)_2_ takes place and the concentration of free lead ions starts to decrease. At pH below and above 8, the predominant species of Cd(ii) is Cd^2+^ and CdOH^+^, Cd(OH)_2_, respectively.

For Pb(ii) and Cd(ii), the removal rate is relatively low at pH 2 and then it increases to 99.0% and 73.5%, respectively, at pH 5(Fig. 5). When the solution pH reaches 8.0, due to the presence of OH^−^, adsorption and precipitation occur simultaneously. The final pH after equilibrium can also be measured, which is higher than the initial value (Fig. 5). On the basis of the analysis, pH 5.0 was selected as the best pH value for further study.

### 3.3 Adsorption kinetics

**Fig. 6:**
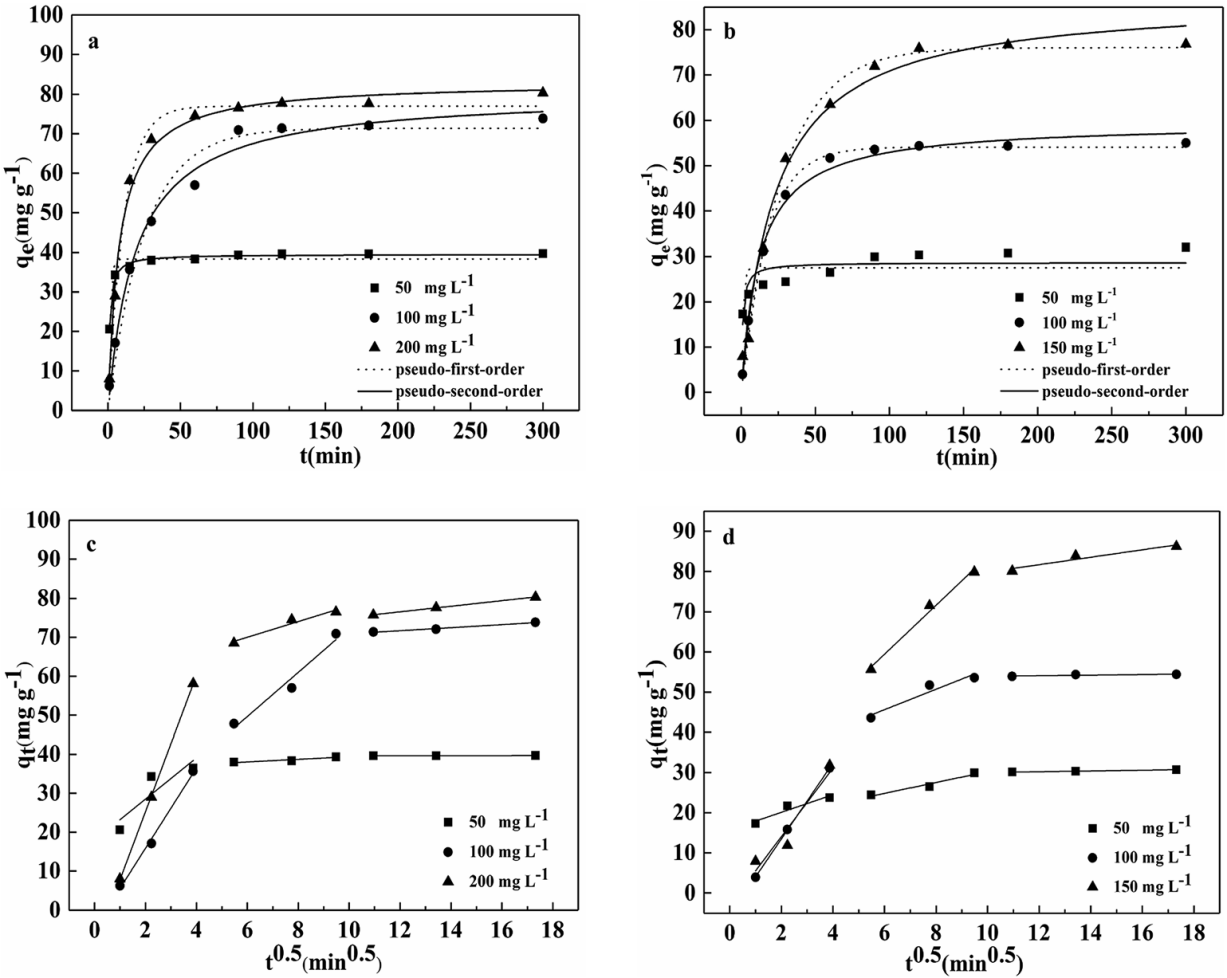
Pseudo-first-order and pseudo-second-order models for (a) Pb(ii) and (b) Cd(ii) on BC/FM, intraparticle diffusion kinetic model for (c) Pb(ii) and (d) Cd(ii) on the BC/FM; pH = 5; temperature = 298 K; BC/FM dose = 1.25 g L^−1^.

The effect of reaction time on Pb(ii) and Cd(ii) removal by BC/FM is shown in Fig. 6a and b. Within the first 30 min, the removal rate of both the metal ions was fast, then dropped off gradually and eventually reached equilibrium at 120 min.

Kinetic study provides vital information about the adsorption mechanism. In order to determine the appropriate sorption kinetics model, three widely used kinetic models, pseudo-first-order kinetics model, pseudo-second-order kinetics model and the intraparticle diffusion model were chosen to describe kinetic experimental data. Their nonlinear equation is as follows:

9 *q*_*t*_ = *q*_e_(1 − e^−*k*_1_*t*^)

10
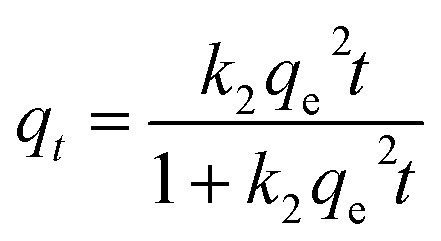


The kinetic model equation of intraparticle diffusion is described as follows:

11 *q*_*t*_ = *k*_ip_*t*^0.5^ + *C*

where *q*_e_ is the equilibrium adsorption capacity (mg g^−1^); *q*_*t*_ is the adsorption capacity at time *t* (mg g^−1^); *k*_1_, *k*_2_, *k*_ip_ are the equilibrium rate constants of the above three models, respectively (min^−1^, mg g^−1^ min, mg g^−1^ min^−0.5^); *t* is the adsorption time (min); *C* is a constant.

The fitting results of the three adsorption kinetic models are shown in Fig. 6, and the parameters calculated are listed in Tables 1 and 2. Evidently, the correlation coefficient (*R*^2^) of the pseudo-second-order kinetics model for both the metal ions was over 0.99 and was greater than that of the pseudo-first-order kinetics model. In addition, for both Pb(ii) and Cd(ii), the theoretical *q*_e_ values calculated from the pseudo-second-order kinetics model is closer to the experimental values(*q*_e,exp_). Thus, these results indicate that the kinetic model of pseudo-second-order is better suited to describe the removal process of Pb(ii) and Cd(ii) by BC/FM and this process is mainly controlled by chemical adsorption.^22,28^

**Table 1:** Kinetic parameters for the adsorption of Pb(ii) and Cd(ii)

Metal	*C*_0_ (mg g^−1^)	Experimental	Pseudo-first-order			Pseudo-second-order		
		*q*_e_ (mg g^−1^)	*q*_e_ (mg g^−1^)	*k*_1_ (min^−1^)	*R* ^2^	*q*_e_ (mg g^−1^)	*k*_2_ (mg g^−1^ min^−1^)	*R* ^2^
Pb	50	39.737	38.369	0.314	0.925	39.533	1.109	0.991
	100	73.846	71.382	0.017	0.974	80.399	0.052	0.990
	200	80.392	77.004	0.039	0.991	83.121	0.130	0.992
Cd	50	32.000	27.520	0.403	0.907	28.677	1.093	0.997
	100	55.024	54.125	0.025	0.996	59.539	0.080	0.992
	150	76.800	76.028	0.015	0.993	76.937	0.044	0.998

**Table 2:** Intraparticle diffusion parameters for the adsorption of Pb(ii) and Cd(ii)

Metal	*C*_0_ (mg g^−1^)	*k*_ip1_ (mg g^−1^ min^−0.5^)	*C*_1_	*R* ^2^	*k*_ip2_ (mg g^−1^ min^−0.5^)	*C*_2_	*R* ^2^	*k*_ip3_ (mg g^−1^ min^−0.5^)	*C*_3_	*R* ^2^
Pb	50	5.294	17.91	0.575	0.334	36.04	0.795	0.009	39.529	0.424
	100	103.800	−4.76	0.990	5.672	15.62	0.926	0.391	67.014	0.980
	200	17.463	−9.68	0.999	2.018	57.92	0.914	0.718	67.946	0.999
Cd	50	1.356	16.70	0.909	2.191	15.73	0.847	0.094	29.109	0.995
	100	2.548	30.32	0.854	9.462	−5.45	0.999	0.072	53.207	0.368
	150	6.108	22.78	0.979	8.530	−3.02	0.837	0.928	70.520	0.859

Fig. 6c and d and Table 2 depict the results of the intraparticle diffusion model fitting for Pb(ii) and Cd(ii) on BC/FM. It indicated that both the metal ions migrated rapidly in the initial stage, then diffused relatively slowly, and finally reached the binding sites of BC/FM. Similar diffusion mode was also studied for Pb(ii) and Cd(ii) removal by HMO-BC^37^ and in other researches.^41,42^ The calculated constant *C* (Table 2) is a non-zero value, implying that the adsorption mechanism of both the metals onto BC/FM is complex and intraparticle diffusion is not the only rate limiting step.

### 3.4 Adsorption isotherm

The study of adsorption isotherm provides vital information, for instance, sorption mechanism. The adsorption isotherm models are fundamental in describing the distribution of adsorbate on adsorbents in the equilibrium state. Freundlich, Langmuir and Sips (Freundlich–Langmuir) adsorption isotherms were employed to explain adsorption equilibrium data in this study. The equations for the three models are provided as follows:12
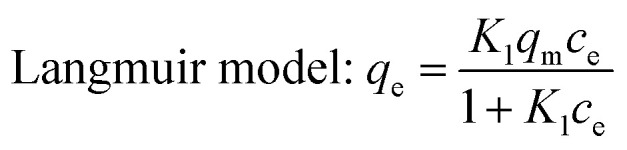

13 Freundlich model: *q*_e_ = *K*_2_*c*_e_^*n*^

14
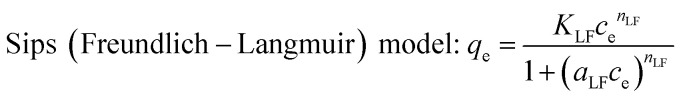
where *c*_e_ is the concentration of Pb(ii) or Cd(ii) at the equilibrium state (mg g^−1^); *q*_e_ is the balanced adsorption capacity of Pb(ii) or Cd(ii) (mg g^−1^); *Q*_m_ indicates the maximum adsorption capacity (mg g^−1^); *K*_1_, *K*_2_ and *K*_LF_, *n*, *n*_LF_ and *a*_LF_ are constants of Langmuir, Freundlich and Sips (Freundlich–Langmuir) model, respectively.

#### 3.4.1 Single solute (Pb or Cd) adsorption isotherms

**Fig. 7:**
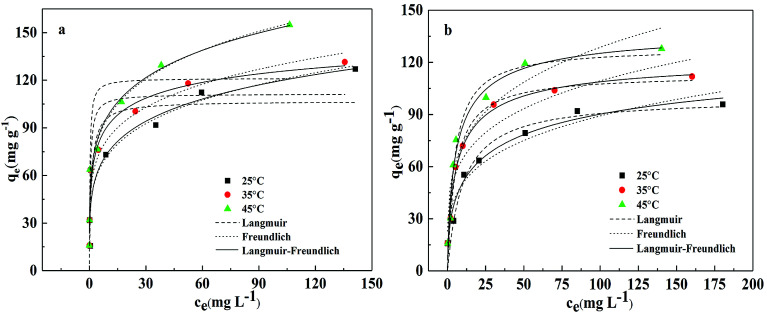
Adsorption isotherm for the adsorption of (a) Pb(ii) and (b) Cd(ii) onto BC/FM in a single solute system; temperatures = 25–45 °C; pH = 5; *t* = 5 h.

The sorption isotherms of both the metals onto BC/FM at three temperatures (25 °C, 35 °C and 45 °C) are displayed in Fig. 7. The adsorption process of both the metal ions onto BC/FM is closely related to temperature, and the adsorption capacity increases with the increase in the solution temperature from 25 °C to 45 °C. This result suggests that the adsorption of both the metal ions onto BC/FM is an endothermic process. The fits to three isotherm models at three different temperatures are depicted in Fig. 7, and the isotherm parameters are given in Table 3.

**Table 3:** Adsorption isotherm parameters for the adsorption of Pb(ii) and Cd(ii) onto BC/FM in a single system

Metal ions	*T* (°C)	Langmuir			Freundlich			Langmuir–Freundlich			
		*Q*_m_ (mg g^−1^)	*K*_1_ (L mg^−1^)	*R* ^2^	*n*	*K*_2_ (mg^1−*n*^ g^−1^ L^−*n*^)	*R* ^2^	*K*_LF_	*a*_LF_	*n*_LF_	*R* ^2^
Pb	25	107.473	0.872	0.785	0.214	44.668	0.891	49.47	2.264	0.260	0.892
	35	111.423	2.077	0.888	0.191	55.822	0.944	83.42	0.144	0.373	0.963
	45	121.290	3.203	0.810	0.183	60.764	0.975	66.07	1.383	0.226	0.976
Cd	25	99.676	0.099	0.941	0.249	28.236	0.942	25.54	0.030	0.487	0.967
	35	113.140	0.191	0.969	0.229	38.101	0.912	32.15	0.15	0.731	0.980
	45	128.532	0.215	0.963	0.245	41.632	0.902	36.37	0.16	0.748	0.972

According to the comparison of the correlation coefficients (*R*^2^), Sips adsorption isotherm model is more suitable for fitting experimental data than the other two models. This result suggests that the removal process of both the metals onto BC/FM is controlled by diffusion and saturated monomolecular adsorption at low and high concentration of Pb(ii) and Cd(ii), respectively.^35^ The experimental maximum adsorption capacity for Pb(ii) and Cd(ii) is 154.94 and 127.83 mg g^−1^, respectively. Compared with some other adsorbents of biochar and biochar-based composites reported in other studies (Table 4), adsorption ability of BC/FM for Pb(ii) and Cd(ii) is greater than most of them. To a certain extent, the high adsorption capacity of both the metal ions on BC/FM can be attributed to the loaded MnFe_2_O_4_. The high affinity of MnFe_2_O_4_ with Pb(ii) and Cd(ii) generated stable inner complexes and complexation of surface functional groups (–COOH, –OH) with metal ions, as reported by Ren *et al.*^26^

**Table 4:** Comparison of the maximum adsorption capacities for Pb(ii) and Cd(ii) onto BC/FM with other adsorbents reported in earlier studies^a^

Adsorbents	*Q*_m_ (mg g^−1^)		Reference
	Pb	Cd	
Biochar from maple wood	43.3	∼	3
Calcium-based magnetic biochar	∼	10.7	13
Manure-derived biochar (DM200/DM350)	∼	31.9/51.4	16
Buffalo weed biochar (BWBC)	333.33	11.63	17
Ferromanganese binary oxide-biochar (FMBC)	∼	101.0	22
Corn straw biochar (BC)	∼	28.0	
Magnetic oak bark biochar	30.20	7.40	35
Magnetic oak wood biochar	10.13	2.87	
Biochar-supported hydrated manganese oxide (BC-HMO)	67.90	22.30	37
Magnetic pine park biochar	25.294	14.960	40
BC/FM	154.94	127.83	This study

a Maximum sorption capacity obtained from sorption experiments.

The separation factor *R*_L_ values calculated by *K*_1_ (*R*_L_ = 1/(1 + *K*_1_*C*_0_)) were all within 0–1, suggesting that the adsorption of both the metals onto BC/FM composite was favorable.^22^ Moreover, the Freundlich constant (*n*) values indicated that Pb(ii) and Cd(ii) is easily adsorbed by BC/FM.^43^

#### 3.4.2 Bi-solute (Pb + Cd) adsorption isotherms

**Fig. 8:**
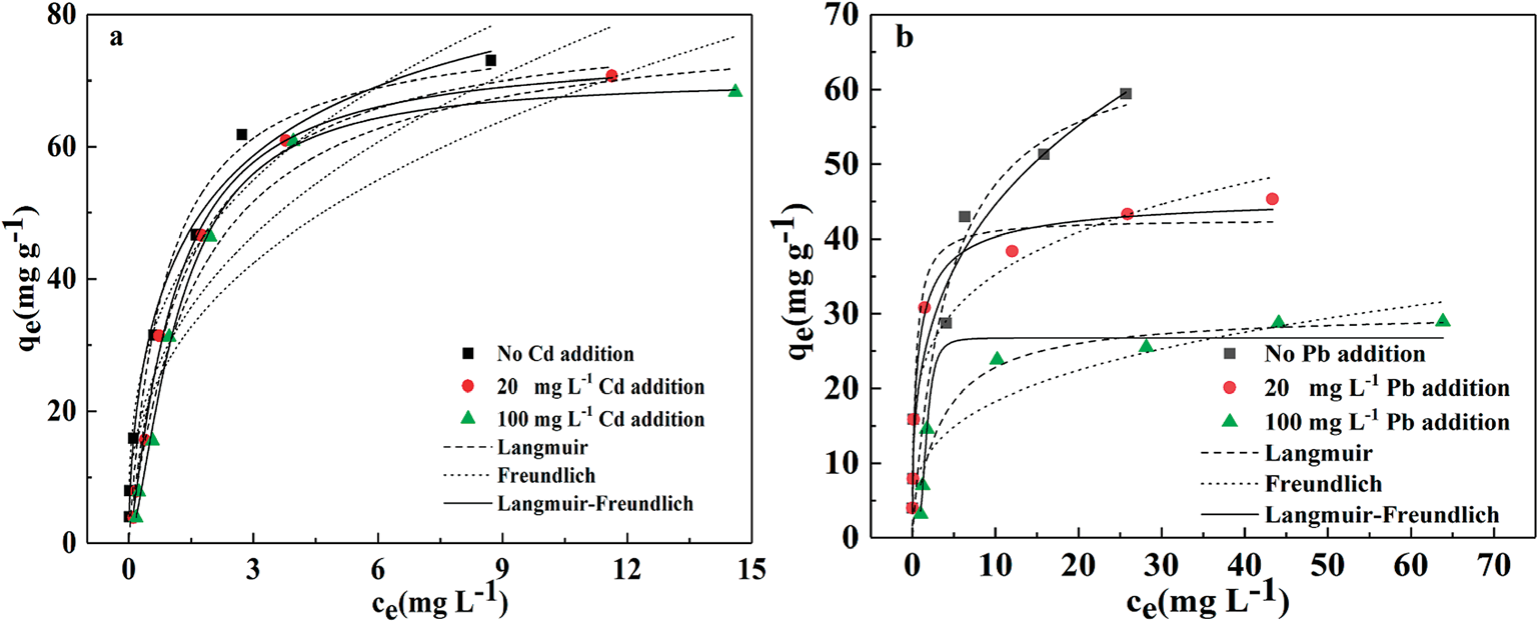
Adsorption isotherm for the adsorption of (a) Pb(ii) and (b) Cd(ii) onto BC/FM in a bi-solute system, temperatures = 25 °C; pH = 5; *t* = 5 h.

Competitive adsorption occurs when two metals coexist in a solution.^44^ In order to understand the mutual effects of the two metal ions, the studies of adsorption by BC/FM in a bi-solute solution were conducted at 25 °C. The sorption isotherms of the two metals in a bi-solute system are shown in Fig. 8 and the corresponding isotherm parameters are given in Table 5.

**Table 5:** Adsorption isotherm parameters for the adsorption of Pb(ii) and Cd(ii) onto BC/FM in bi-solute system

System	Langmuir			Freundlich			Sips (Langmuir–Freundlich)			
	*Q*_m_ (mg g^−1^)	*K*_1_ (L mg^−1^)	*R* ^2^	*n*	*K*_2_ (mg^1−*n*^ g^−1^ L^−*n*^)	*R* ^2^	*K*_LF_	*a*_LF_	*n*_LF_	*R* ^2^
Pb (no Cd addition)	78.780	1.166	0.971	0.331	38.188	0.969	67.829	0.479	0.604	0.991
Pb + Cd (20 mg L^−1)^	80.096	0.769	0.993	0.382	30.598	0.896	68.621	0.934	1.197	0.997
Pb + Cd (100 mg L^−1^)	79.801	0.613	0.977	0.374	28.132	0.843	55.684	0.833	1.480	0.997
Cd (no Pb addition)	67.679	0.231	0.897	0.308	21.913	0.973	21.913	1.837	0.308	0.973
Cd + Pb (20 mg L^−1^)	42.628	2.562	0.973	0.214	21.527	0.930	64.577	1.631	0.676	0.985
Cd + Pb (100 mg L^−1^)	30.266	0.305	0.945	0.295	9.254	0.858	4.082	0.596	3.633	0.971

For Pb(ii), the adsorption capacity was hardly affected by the addition of Cd(ii) at 20 and 100 mg L^−1^ (Fig. 8a). In comparison, the adsorption of Cd(ii) decreased markedly by the addition of Pb(ii) and the effect was greater at 100 mg Pb L^−1^ than 20 mg Pb L^−1^. The Langmuir adsorption capacity of Cd(ii) decreased to 42.628 and 30.266 mg g^−1^, respectively (Fig. 8b), demonstrating the competition for the binding sites between them. To summarize, the preferential adsorption order of BC/FM for the two metals is Pb(ii) > Cd(ii).

The relative adsorption for metal ions is mainly related to their ionic radius, ionic potential, hydrolysis state and chemical properties.^35^ The larger Pb^2+^ ion size (1.32 Å) *versus* Cd^2+^ ion (1.03 Å) may be connected to the higher Pb(ii) adsorption.^35^ The ionic potential (ionic charge/radius ratio) of Pb^2+^ ion and Cd^2+^ ion is 3.3 and 1.9 respectively, which leads to stronger Pb^2+^ ionic interaction force *versus* Cd^2+^, thus Pb^2+^ will be adsorbed preferentially. With respect to hydration energy, Pb(ii) (−1504 kJ mol^−1^) has lower hydration energy than Cd(ii) (−1708 kJ mol^−1^), which indicates that Pb(ii) is more favorable for ion exchange with BC/FM.^37^ As for other aspects, the complex nature of BC/FM composite and the abundant functional groups also play a vital role in the relative adsorption.

### 3.5 Adsorption thermodynamics

To reveal the adsorption thermodynamic characteristic of the two metals by the prepared BC/FM, the Gibbs free energy (Δ*G*°), enthalpy change (Δ*H*°), and entropy change (Δ*S*°) of sorption were calculated using the van't Hoff thermodynamic equations:

15 Δ*G*° = −*RT* ln *K*_C_

16 *K*_C_ = *q*_e_/*C*_e_

17 ln *K*_C_ = Δ*S*°/*R* − Δ*H*°/(*RT*)

where *T* is the absolute temperature (K) and *R* is the universal gas constant (8.314 J mol^−1^ K^−1^). The thermodynamic parameters obtained are given in Table 6. Δ*G*° < 0 implies that the adsorption process of both the metal ions on BC/FM is spontaneous, while Δ*H*° > 0 indicates that the process is endothermic and is favorable at higher temperatures. Δ*S*° > 0 implies the increase in randomness owing to the adsorption of Pb(ii) and Cd(ii) from the solution. Therefore, the above results show that the adsorption process in this study was mainly chemisorption.

**Table 6:** Thermodynamic parameters of Pb(ii) and Cd(ii) adsorption onto BC/FM

Metal ions	*T* (K)	ln *K*_LF_ (kJ mol^−1^)	Δ*G* (kJ mol^−1^)	Δ*H* (kJ mol^−1^)	Δ*S* (J mol^−1^ K^−1^)
Pb	298.15	3.901	−9.671	27.993	125.541
	308.15	4.424	−11.334		
	318.15	4.191	−11.085		
Cd	298.15	3.240	−8.032	16.146	81.003
	308.15	3.470	−8.891		
	318.15	3.594	−9.506		

### 3.6 Sorption mechanism

#### 3.6.1 FTIR analysis

**Fig. 9:**
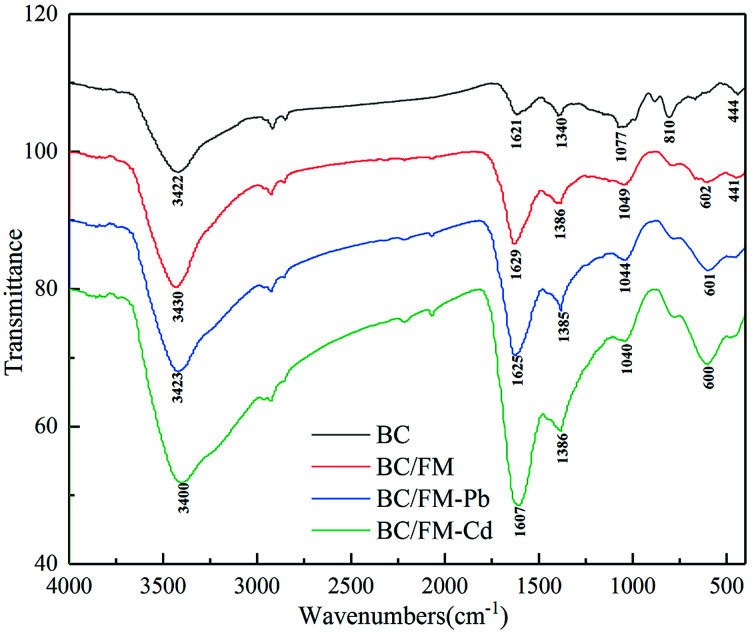
FTIR spectra of BC, BC/FM, BC/FM-Pb, and BC/FM-Cd.

The FTIR spectra of BC, BC/FM, BC/FM-Pb, and BC/FM-Cd samples are shown in Fig. 9. The broad band around 3400 cm^−1^ can be attributed to –OH group stretching vibration. The peaks at 2922 and 2852 cm^−1^ are ascribed to –CH_2_ groups. Compared with pristine BC, a new peak appears at 602 cm^−1^ on BC/FM, which could be ascribed to Fe–O,^26^ typical of spinel ferrite, but it moves to 601 cm^−1^ and 600 cm^−1^ after Pb(ii) and Cd(ii) adsorption, respectively. The broad adsorption peaks around 1629 and 1386 cm^−1^ represent the stretching vibrations of C=C and carboxyl C=O.^45,46^ Nevertheless, such peaks evidently move to a lower frequency at about 1625 and 1385 cm^−1^ for BC/FM-Pb, and to 1607 and 1386 cm^−1^ for BC/FM-Cd. The band at 1049 cm^−1^ is attributed to C–O stretching vibrations, but it shifts to 1044 and 1040 cm^−1^ for BC/FM-Pb and BC/FM-Cd, respectively. Generally, the results suggested that the surface functional groups of BC/FM undergo qualitative changes before and after adsorption. It indicated that the hydroxyl and the carboxyl groups were used up mostly in the process of Pb(ii) and Cd(ii) adsorption to form inner complexes (–COO–M, –O–M and Fe–O–M (M = Pb or Cd)), which lead to the displacement of the characteristic band of COOH and O–H. Furthermore, these spectra provided proof for strong interactions between loaded MnFe_2_O_4_ and Pb/Cd. Similarly, the significant shift of hydroxyl groups of ferromanganese binary oxide-biochar composites after Cd(ii) and Cu(ii) adsorption were reported.^22^

#### 3.6.2 XPS analysis

**Fig. 10:**
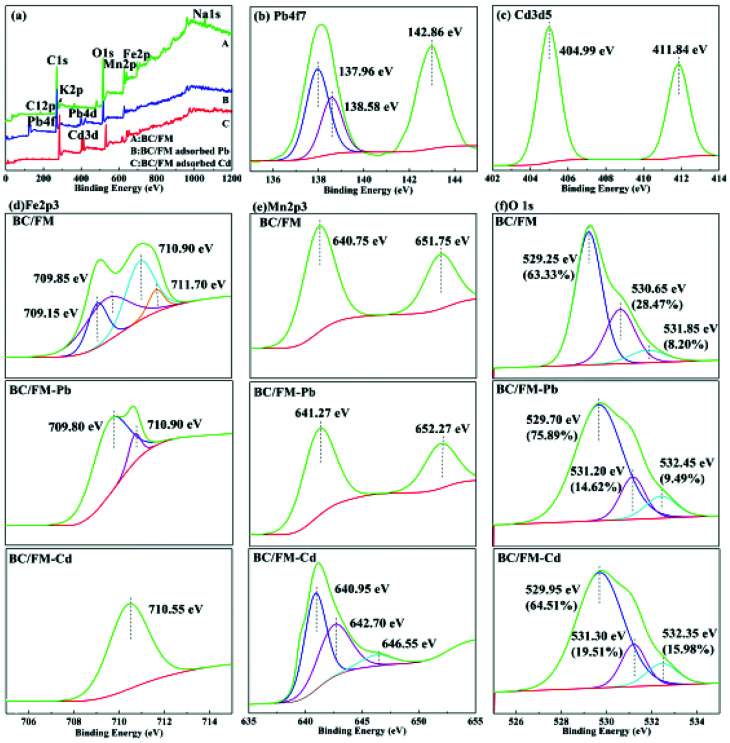
XPS spectra of BC/FM before and after adsorption for Pb(ii) and Cd(ii). (a) XPS spectra of BC/FM before and after Pb(ii) and Cd(ii) adsorption; (b) Pb 4f XPS spectra of BC/FM-Pb; (c) Cd 3d XPS spectra of BC/FM-Cd; (d) Fe 2p XPS spectra of BC/FM, BC/FM-Pb and BC/FM-Cd; (e) Mn 2p XPS spectra of BC/FM, BC/FM-Pb and BC/FM-Cd; (f) O 1s XPS spectra of BC/FM, BC/FM-Pb and BC/FM-Cd.

In order to further investigate the removal mechanism of Pb(ii) and Cd(ii) by BC/FM, XPS analysis on BC/FM and Pb/Cd-BC/FM were performed (Fig. 10). Fig. 10a shows that elements K and Na existed in BC/FM, but disappeared after Pb(ii) or Cd(ii) adsorption. Since Na and K have strong ion exchange ability, it proves the existence of the ion exchange reaction in Pb(ii) and Cd(ii) removal process. The Mn 2p^3^ and Fe 2p^3^ peaks are found on BC/FM surface before and after Pb/Cd adsorption. The significant peaks corresponding to Pb 4f and Cd 3d were observed on BC/FM surface after Pb and Cd were adsorbed, respectively.

The species of Pb and Cd adsorbed on BC/FM surface was also analyzed (Fig. 10b and c). XPS of Pb 4f in Fig. 10b showed a significant shift to a lower binding energy for 4f_7/2_ at 138.23 eV in comparison with Pb(NO_3_)_2_ that was centered at 139.5 eV for 4f_7/2_. The results indicate that not a mere electrostatic interaction but a specific sorption could explain the mechanism of Pb adsorption onto BC/FM, and the interaction was attributed to Pb–O binding or hydroxyl and carboxyl binding. Binding energy of Cd 3d_3/2_ and Cd 3d_5/2_ appears at 411.84 eV and 404.99 eV, respectively. The interaction between Cd and BC or MnFe_2_O_4_ was explained by Cd-hydroxyl and Cd-carboxyl bond formed with the BC/FM surface.

Detailed spectra of Fe 2p and Mn 2p are shown in Fig. 10d and e. The binding energy of 709 and 712 eV of Fe 2p_3/2_ have a bearing on Fe 2p and Fe 3p, respectively.^22^ The binding energies of 709.8 and 710.9 eV (FeOOH and FeO), and 710.5 eV of Fe 2p_3/2_ imply that both Fe 2p and Fe 3p exist. The Mn 2p_3/2_ peak at 640.75 eV and the Mn 2p_1/2_ peak at 651.75 eV were also found. The interval of 11.0 eV implied that the predominant Mn oxidation state in BC/FM was Mn^2+^. It could be noted that there is a shift in Mn 2p_3/2_ line toward higher binding energies in case of BC/FM-Pb and BC/FM-Cd. Especially for BC/FM-Cd, a peak at 640.95 and 642.70 eV for Mn 2p_3/2_ suggests the presence of Mn_2_O_3_ and MnO_2_.

The O 1 s spectrum (Fig. 10f) was deconvoluted into three peaks, located at 529.25, 530.65, and 531.85 eV, which can be ascribed to Fe–O, C–OH/C–O, and Mn–O, respectively.^22,45^ We calculated the ratio of Fe–O and Mn–O peak areas grown after Pb (Fe–O, 63.33–75.89%; Mn–O, 8.20–9.49%) or Cd (Fe–O, 63.33–64.51%; Mn–O, 8.20–15.98%) adsorption. In contrast, the ratio of C–O/C–OH peak areas reduced from 28.47% to 14.62% or 19.51% after adsorption of Pb or Cd, respectively. To the best our knowledge, the increase in Fe–O and Mn–O is ascribed to the formation of M–O–R (M denote Fe or Mn; R denote functional groups), Pb–O or Cd–O group on the surface of BC/FM after adsorption. The prominent reduction of oxygen for C–OH/C–O indicates that hydroxyl and carboxyl groups of BC surely participate in the adsorption process.

These results suggest that there is a strong complexation between Pb(ii), Cd(ii) and MnFe_2_O_4_ as well as oxygen-containing functional groups on BC/FM, and the complexation played a vital role in the removal process of Pb(ii) and Cd(ii) by BC/FM. In addition, the role of the ion exchange reaction can not be ignored in this process.

## 4. Conclusion

A functionalized biochar-supported magnetic MnFe_2_O_4_ nanocomposite (BC/FM) was developed through sol–gel process using egg white and pyrolysis at 500 °C. The SEM-EDX, BET, XRD, FTIR and XPS analyses show that the magnetic MnFe_2_O_4_ adhere onto the surface of the biochar, and Pb(ii) and Cd(ii) were absorbed by BC/FM composite successfully. The results of adsorption experiments indicate that the removal process for Pb(ii) and Cd(ii) in a single-solution is pH-dependent. The adsorption preference of the two metals by BC/FM in bi-solute system was Pb(ii) prior to Cd(ii) and the competition effects existed. The BC/FM composite exhibited an outstanding adsorption ability towards both the metals in the system of single or double solute. XPS and FTIR analysis verified that the main removal mechanism of Pb(ii) and Cd(ii) by BC/FM was the formation of Pb/Cd–O or complexation of carboxyl and hydroxyl with both the metals ions and ion exchange. Thus, BC/FM composite has been proved to be an outstanding adsorbent for Pb(ii) and Cd(ii) removal from wastewater.

## Conflicts of interest

There are no conflicts to declare.

## Supplementary Material
